# Effect of Exo/Endogenous Prophylaxis Dentifrice/Drug and Cariogenic Conditions of Patient on Molecular Property of Dental Biofilm: Synchrotron FTIR Spectroscopic Study

**DOI:** 10.3390/pharmaceutics14071355

**Published:** 2022-06-26

**Authors:** Pavel Seredin, Dmitry Goloshchapov, Vladimir Kashkarov, Dmitry Nesterov, Yuri Ippolitov, Ivan Ippolitov, Jitraporn Vongsvivut

**Affiliations:** 1 Department of Solid-State Physics and Nanostructures, Voronezh State University, 394018 Voronezh, Russia; goloshchapovdl@gmail.com (D.G.); kash@phys.vsu.ru (V.K.); nesterov@phys.vsu.ru (D.N.); 2 Scientific and Educational Center “Nanomaterials and Nanotechnologies”, Ural Federal University, 19 Mira Street, 620002 Ekaterinburg, Russia; 3 Department of Pediatric Dentistry with Orthodontia, Voronezh State Medical University, 394006 Voronezh, Russia; dsvgma@mail.ru (Y.I.); stomat@vmail.ru (I.I.); 4 Australian Synchrotron, Clayton, VIC 3168, Australia; jitrapov@ansto.gov.au

**Keywords:** dental biofilm and tissue, biomimetics, molecular properties, endo- and exogenous prophylaxis, infrared microspectroscopy, synchrotron radiation

## Abstract

(1) Objectives: This study is the first one to investigate the molecular composition of the dental biofilm during the exogenous and endogenous prophylaxis stages (use of dentifrice/drug) of individuals with different cariogenic conditions using molecular spectroscopy methods. (2) Materials and Methods: The study involved 100 participants (50 males and 50 females), aged 18–25 years with different caries conditions. Biofilm samples were collected from the teeth surface of all participants. The molecular composition of biofilms was investigated using synchrotron infrared microspectroscopy. Changes in the molecular composition were studied through calculation and analysis of ratios between organic and mineral components of biofilm samples. (3) Results: Based on the data obtained by synchrotron FTIR, calculations of organic and mineral component ratios, and statistical analysis of the data, we were able to assess changes occurring in the molecular composition of the dental biofilm. Variations in the phosphate/protein/lipid, phosphate/mineral, and phospholipid/lipid ratios and the presence of statistically significant intra- and inter-group differences in these ratios indicate that the mechanisms of ion adsorption, compounds and complexes arriving from oral fluid into dental biofilm during exo/endogenous prophylaxis, differ for patients in norm and caries development. (4) Conclusions: The conformational environment and charge interaction in the microbiota and the electrostatic state of the biofilm protein network in patients with different cariogenic conditions play an important role. (5) Clinical Significance: Understanding the changes that occur in the molecular composition of the dental biofilm in different oral homeostasis conditions will enable successful transition to a personalised approach in dentistry and high-tech healthcare.

## 1. Introduction

In recent years, dental biofilm has been the subject of active research, as it is involved in the development of caries and chronic and acute infections [1,2,3,4,5], and in metabolic processes in the oral cavity [6,7,8,9]. The conditions of these physico-chemical processes depend on the composition and properties of the dental biofilm [6,8,10,11,12], including various organic substances and mineral ions, bacteria, and water [3,6,11,13,14].

The biofilm protein network carries many different ions and molecules, and binds and contributes to their oversaturation with respect to the enamel mineral complex [6,15,16,17]. The biofilm protein network contacts the enamel surface, the organic matrix, the enamel rods, and the hydrate layer on the surface of hydroxyapatite nanocrystals, through which all metabolic processes occur at the enamel apatite crystal boundary [6,18] and also form on biomimetic restorative materials, such as resin-based composites with different degrees of finishing due to their roughness [19,20,21]. The biofilm directly and indirectly comes into contact with the oral fluid, which contains the ions and complexes required for enamel remineralisation [9,17]. Introduction into the oral cavity and subsequent accumulation in the biofilm of substances capable of forming chemically stable compounds in the enamel surface layer are tasks included in remineralising therapy, which can be performed by exogenous and endogenous methods [9,22,23,24,25].

It has been demonstrated in our previous studies that exogenous and endogenous prophylaxis methods have different effects on the molecular composition and organo-mineral balance of the oral fluid [26,27]. Endogenous prophylaxis methods result in the long-term presence of minerals and organo-mineral groups and complexes required for enamel remineralisation in the oral fluid, which is a prerequisite for their increased concentration in the biofilm and thus on the enamel surface [27]. It is clear that the effective management of hard tissue mineralisation processes requires new strategies for identifying and controlling the molecular composition of the dental biofilm.

The most convenient and sought-after approach is Fourier transform infrared spectroscopy (FTIR) [28,29,30,31]. The use of FTIR in biofilm analysis has shown promising results, particularly for the detection and identification of biofilm-forming bacteria [28,29,32,33]. Furthermore, an undeniable advantage of FTIR over genetic assays is that the latter can provide information that is not always consistent with cellular phenotypes [28,29,32,33], whereas FTIR allows reliable tracking of molecular biochemical changes occurring in the analyte over time [29,33].

Unfortunately, in the vast body of data already available, there is no information obtained by FTIR on the molecular composition of the dental biofilm of human teeth in normal patients or in the development of carious pathologies, or on changes occurring in the biofilm using different prophylactic agents. One plausible explanation is that the microorganisms that make up the dental biofilm aggregate into complex microbial communities that function differently from those of planktonic cells [3,10,13,34]. Microorganisms that make up the dental biofilm have often been studied in the planktonic state using optical and electron microscopy methods [32,34].

Thus, the aim of our study is to investigate the molecular composition of the dental biofilm during exogenous and endogenous prophylaxis stages in individuals with different cariogenic conditions using synchrotron infrared molecular spectroscopy techniques.

## 2. Materials and Methods

### 2.1. Research Design

The study involved 100 participants (50 males and 50 females) who were aged 18–25 years, Caucasian, physically healthy, without bad habits, and with different caries conditions.

Participants in the first (healthy) group (25 men and 25 women) had no clinically detectable dental caries lesions. Participants in the second (carious) group (25 men and 25 women) had teeth with surface caries (ICDAS 1–2 [35]). There were no signs of periodontitis or gingivitis in any of the participants.

For the week preceding the experiment, the patients ate mainly plant-based foods, followed a standard water regime, did not take any medication, and did not drink alcohol.

Biofilm samples were collected from the teeth surface of all participants. The biofilm was carefully removed from the surface of the maxillary central incisors using a sterile scalpel, without touching the gingival sulcus.

The procedure for obtaining dental biofilm samples in our study was performed as follows. In stage I, biofilm samples were collected from study participants on the eighth day after the start of observation, with no change in oral hygiene conditions. Collection took place in the morning before oral hygiene with toothpaste and meals. Thirty minutes after mechanical brushing with a soft toothbrush for 3 min (to remove dental plaque residues from the tooth surface) and a preliminary rinse with clean water, biofilm was collected from the two groups of study participants for the first time.

In stage II of the experiment (the next day), participants used a toothpaste with calcium glycerophosphate to clean their teeth [36,37]. Thirty minutes after oral hygiene and rinsing with clean water, biofilm was collected from the two groups of study participants for a second time.

The next day, after a meal, participants began taking tablets containing a mineral complex with calcium glycerophosphate [36,37]. Participants took one tablet, three times per day. On the fourth day, in the morning before meals, 30 min after mechanical cleaning of the teeth with a soft toothbrush for 3 min (to remove dental plaque residues from the tooth surface) and a preliminary rinse with clean water, biofilm was collected from the two groups of study participants for a third time (stage III).

After collection, biofilm samples were stored at 4 °C.

### 2.2. Equipment Setup and Sample Scanning

The molecular composition of biofilms was investigated using infrared microspectroscopy (IRM) beamline equipment (Australian synchrotron, Clayton, VIC, Australia) using a Bruker Vertex 80v spectrometer coupled with a Hyperion 2000 FTIR microscope and a liquid nitrogen-cooled narrow-band mercury cadmium telluride (MCT) detector (Bruker Optik GmbH, Ettlingen, Germany). All synchrotron FTIR spectra were recorded within a spectral range of 3800–700 cm^−1^ using 4 cm^−1^ spectral resolution. Blackman–Harris three-term apodization, Mertz phase correction, and a zero-filling factor of 2 were set as default acquisition parameters using the OPUS 7.2 software suite (Bruker Optik GmbH, Ettlingen, Germany) [33,38,39].

For synchrotron measurement of FTIR transmission, small pieces of powdered sample were transferred and pressed between a pair of diamond microcompression cell glasses (Thermo Fisher Scientific, Scoresby, VIC, Australia), together with a small piece of KBr powder used as a reference IR background. Spectral data were collected in transmission mode using lens 36 (NA = 0.50; Bruker Optik GmbH, Ettlingen, Germany), a beam focus diameter of 6.9 µm, and eight co-directional scans per spectrum [40]. Background spectra were obtained on KBr, which was well-separated from the powdered sample inside the diamond compression cell using 32 combined scans.

### 2.3. Study Design Scheme

**Figure 1:**
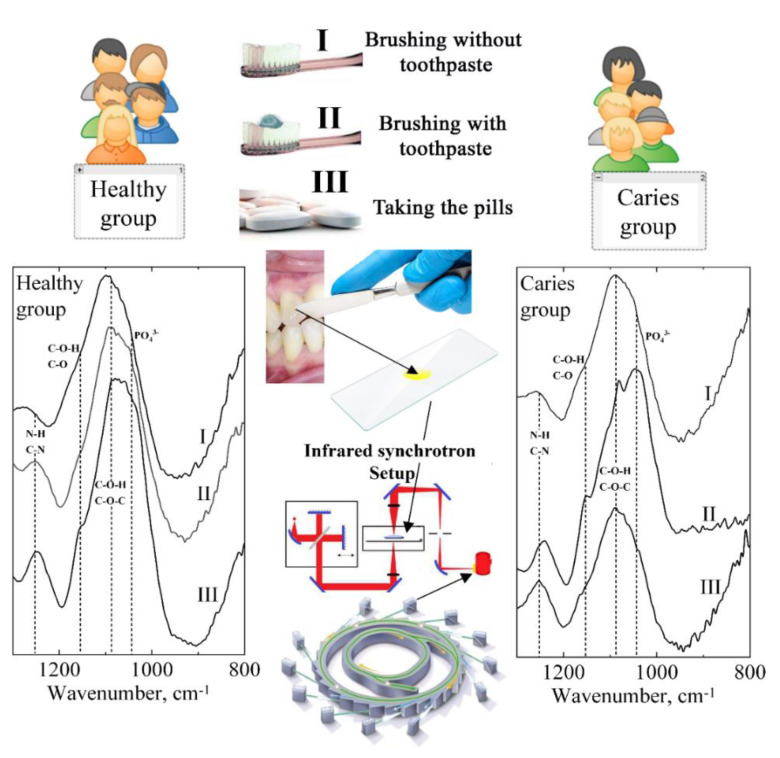
Schematic representation of experimental design.

The design of our study is shown in Figure 1.

### 2.4. Statistical Analysis

Statistical analysis of the results was performed using the professional SPSS software package, ver. 19 for Windows (SPSS Inc., Chicago, IL, USA). Descriptive statistics in the groups were obtained using a standard *t*-test and presented as mean ± standard deviation. Statistical analysis of intergroup differences between participants was performed using nonparametric ANOVA analysis of variance based on Kruskal–Wallis one-way analysis. Duncan’s multiple comparison test was used to determine the significance of the effect of prophylaxis type used by each group of participants in the experiment (intragroup differences).

## 3. Experimental Results

**Figure 2:**
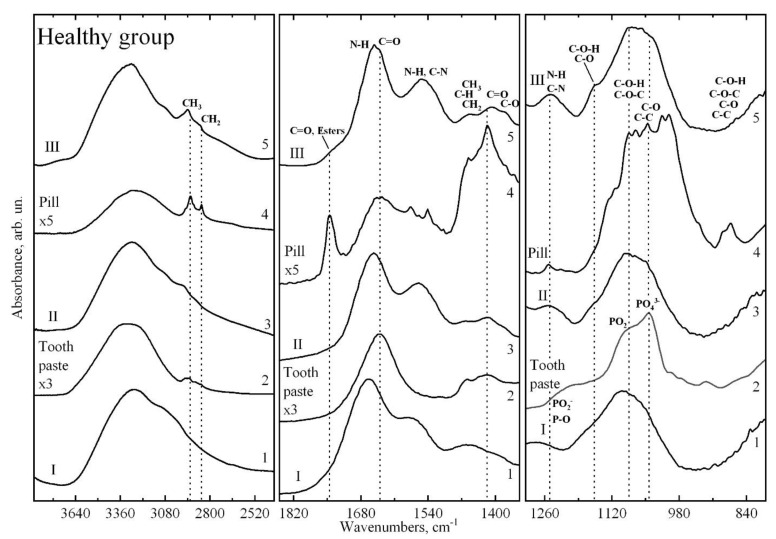
Infrared absorption spectra of dental biofilm samples from healthy group in different stages of the study. 1, FTIR spectra of dental biofilm at stage I; 2, FTIR spectra of tooth paste; 3, FTIR spectra of dental biofilm at stage II; 4, FTIR spectra of the pill with calcium glycerophosphate mineral complex; 5, FTIR spectra of dental biofilm at stage III.

**Figure 3:**
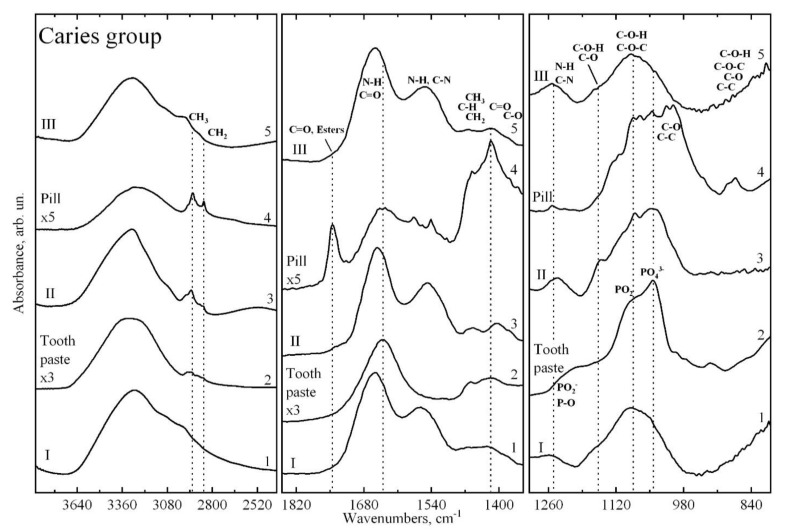
Infrared absorption spectra of carious dental biofilm samples in different stages of the study. 1, FTIR spectra of dental biofilm at stage I; 2, FTIR spectra of tooth paste; 3, FTIR spectra of dental biofilm at stage II; 4, FTIR spectra of the pill with calcium glycerophosphate mineral complex; 5, FTIR spectra of dental biofilm at stage III.

Figure 2 and Figure 3 show the infrared absorption spectra of dental biofilm samples collected from two groups of participants with different cariogenic conditions at different stages of the study. The spectra were obtained using a high-pressure diamond prism attached to an IR microscope and synchrotron radiation [40]. Preliminary consideration of the results indicates that at a particular stage of the experiment, the spectra for the two groups of participants contain the same set of vibrational modes corresponding to characteristic molecular bonds. The spectra in a particular sample differ insignificantly from each other in intensity, due to the individual characteristics of the participants in the experiment. As such, Figure 2 and Figure 3 present the infrared absorption spectra of the dental biofilm before prophylaxis (stage I), after toothpaste (stage II), and after calcium glycerophosphate mineral complex (stage III), averaged over the groups participating in the study.

Analysis of the obtained spectra, based on studies in which IR spectroscopy was used to study oral biological fluids, biofilms, human dental hard tissues, and phosphates relevant to enamel and dentin formation [26,28,29,41,42,43,44,45,46,47], showed that the experimental IR spectra of biofilms have a typical set of basic vibrations.

The active vibrations in the IR spectra, their frequencies, and their molecular group affiliation are presented in Table 1. The main absorption bands present in all IR spectra of biofilm samples can be associated with the following molecular groups.

The broad vibrational band at 3600–3100 cm^−1^ corresponds to the N–H bonds of proteins and may also be related to the presence of O-H hydroxyl groups (i.e., water) in the samples [28,29,42]. The group of bands localised in the range of 2950–2750 cm^−1^ corresponds to vibrations of C–H bonds of various fatty acids and lipids [28,29]. The intensities of these bands in the infrared spectra of biofilms were observed for groups with different cariogenic conditions (Figure 2 and Figure 3).

A band of IR spectra from 1850 to 1350 cm^−1^ includes a band in the region of 1730 cm^−1^ that can be attributed to (>C=O) phospholipids, esters, fatty acids [28,29,43,48], and the characteristic region of proteins. Protein bands include Amide I (C=O stretch) vibrations in the region from 1675–1615 cm^−1^, Amide II (60% N–H bend and 40% C–N stretch) vibrations in the region from 1575–1520 cm^−1^, and Amide III (40% C–N stretch, 30% N–H bend) vibrations in the region from 1315–1270 cm^−1^ [28,29,41,42,43,45]. For dental biofilm, these vibrations can also be correlated with peptides [28,43]. The distinguished group of bands in the region from 1480 to 1350 cm^−1^ correlate with vibrations of CH_2_/CH_3_ groups of proteins and lipids [28,29,41,45]. The band at 1394 cm^−1^ represents a C=O symmetric stretch of the COO- group and is related to biofilm lipids [28,29,41,45]. In the range from 1300–800 cm^−1^, a group of high-intensity vibrations associated with phosphorus derivatives such as phosphates, glycerophosphates, and phospholipids is observed [28,29,44].

In addition to these molecular group vibration modes, there are bands in the IR absorption spectra of biofilms whose appearance and intensity depend on both the cariogenic conditions and the stage of the experiment (the type of preventive measures). Such modes in the spectrum should include the shoulder modes in the regions of 1235 cm^−1^ and 1082 cm^−1^ associated with PO^−2^ asymmetric and symmetric valence vibrations of phosphate residues and phospholipids [28,29,44]. A mode located in the region from 1065 to 1050 cm^−1^ represents overlapping bands of vibrations associated with organic phosphate derivatives, glycerophosphate and phosphatase, the C–O–P–O–C complex, and cellular carbohydrate.

The low-intensity vibrations observed in the region from 900 to 800 cm^−1^ represent a cluster of bands referred to as the “fingerprint region” for protein fractions and bacteria associated with anomeric ring vibrations for tryptophan, tyrosine, and phenyloalanine [28,29].

**Figure 4:**
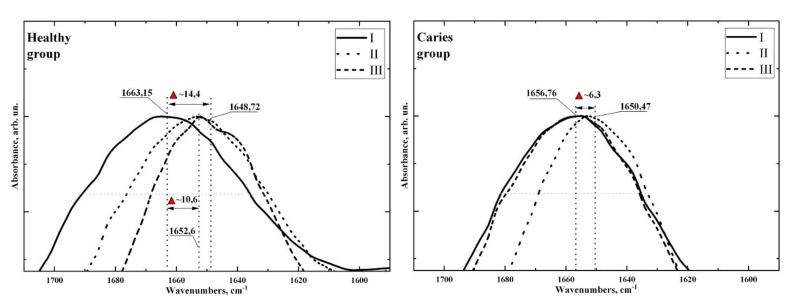
Amide I band profile in IR spectra of healthy (**left**) and carious (**right**) groups.

In Figure 2 and Figure 3, together with the spectra of dental biofilm samples, we show the infrared absorption spectra of the toothpaste with calcium glycerophosphate used in the second stage of the experiment and the tablet containing the mineral complex with calcium glycerophosphate used in the third stage of the experiment. Comparing the spectra of the prophylactics with the spectra of the dental biofilm shows that the IR spectra of the biofilm at the respective stages of the experiment contain specific groups of vibrations characteristic of the IR absorption spectra of the prophylactics, due to the presence of different organomineral complexes in the prophylactic agents whose molecular composition is similar to that of the biofilm organomineral complex. From this, we can draw a preliminary conclusion that the use of prophylactics at the appropriate stage of the experiment can affect the molecular composition of biofilms [9,37], which in turn is reflected in the spectroscopic characteristics. It is clearly observed that the effect of the prophylactic agent depending on the cariogenic conditions is reflected in the position and shape of the vibrational mode of Amide I (Figure 4).

Thus, for the first (healthy) group, the use of toothpaste and tablets led to a significant (up to 14 cm^−1^) shift of the Amide band to the low-frequency region in relation to its position in stage I of the experiment (without prophylactics). For the second (carious) group, a low-frequency (up to 6 cm^−1^) shift for the Amide I band was recorded only in stage II (use of toothpaste); there was no shift for stage III (use of tablet).

In our previous studies [26,27], changes in the molecular composition of biological fluids in the oral cavity were observed, including in the development of pathology. Mathematical estimation of these changes was possible through calculation and analysis of ratios (factors) between organic and mineral components of a biofilm sample. This can also be achieved using the ratio of the intensities of the vibrational bands associated with specific molecular groups. Following the logic of our previous studies [26,27], we calculated several such ratios; we describe only those that are significant, as in our opinion they reflect the essence of the changes occurring in the molecular composition of the dental biofilm.

The R1 mineral–organic ratio (phosphate/protein/lipid) is calculated from the ratio of the integrated band intensity in the region from 1110 to 960 cm^−1^ related to mineral component and phosphate derivatives, the total integrated intensity of the Amide I (C=O stretching) and Amide II bands (CN stretching, NH bending) in the region from 1720 to 1458 cm^−1^ (proteins), and the integrated intensity of the CH_2_/CH_3_ bond vibration bands localised at 1430–1360 cm^−1^ (lipids).

The R2 phosphate/mineral ratio is calculated from the ratio of the total integral intensity of the PO^−2^ vibrational band, with its maximum at 1250–1240 cm^−1^, associated with phosphate residues and phospholipids and glycerophosphate vibrations located near 1060–1030 cm^−1^, to the integral intensity of the band at 1110–960 cm^−1^, correlated with the mineral component.

The R3 phospholipid/lipid ratio is calculated from the ratio of the integral intensity of CH_2_ and CH_3_ vibrations of the phospholipid and fatty acid groups located in the range from 2990 to 2840 cm^−1^ to the integral intensity of CH_2_ and CH_3_ bonds of lipids in the range from 1490 to 1360 cm^−1^.

**Figure 5:**
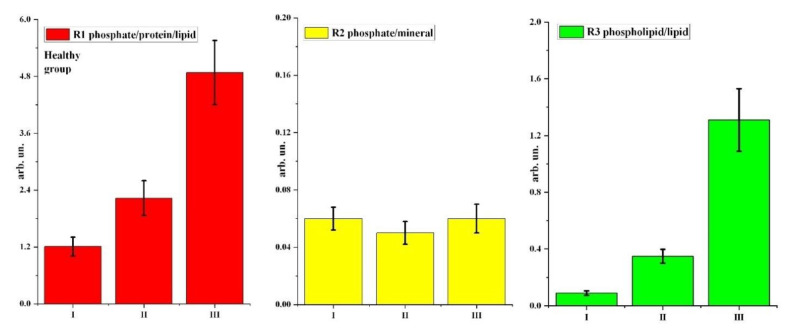
Bar chart diagrams of changes in R1–R3 ratios at different stages of the experiment for the healthy group. Error bars represent one standard deviation (see Table 2).

**Figure 6:**
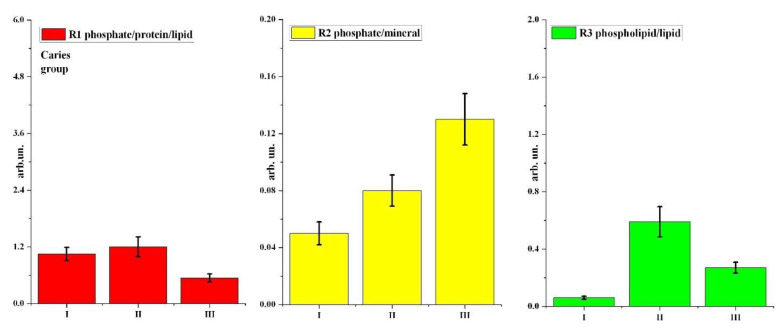
Bar chart diagrams of changes in R1–R3 ratios at different stages of the experiment for the carious group. Error bars represent one standard deviation (see Table 2).

We calculated these ratios for two groups of participants in stages I, II, and III of the study using OPUS 7.2 software (Bruker), which includes a wide range of functionalities for processing and evaluating data obtained by infrared spectroscopy methods. The results of R1–R3 ratio calculations and descriptive statistics data are shown in Table 2 and Figure 5 and Figure 6.

To determine the effect of prophylaxis type (exo/endo) on the molecular composition of the biofilm for the participant groups (healthy/carious), significant intragroup differences between the R1–R3 ratios were determined. We compared R1–R3 ratios using Duncan’s multiple comparison test for each participant group relative to Stage I (without prophylaxis) when using toothpaste and calcium glycerophosphate tablets. Statistically significant intragroup differences at the *p* < 0.05 significance level are indicated in Table 3.

Statistically significant intergroup differences for R1–R3 ratios were determined from analysis of variance. Using a non-parametric Kruskal–Wallis one-way analysis for each prophylaxis type, we compared R1–R3 ratios in the healthy and carious groups in pairs. Statistically significant differences at the *p* < 0.05 significance level are shown in Table 4.

## 4. Discussion

From the results obtained using synchrotron FTIR, calculations of the ratios between organic and mineral components, and statistical data analysis, we were able to assess changes in the molecular composition of the dental biofilm during the exo and endogenous prevention stages for the two groups of study participants (healthy and carious).

Analysing the results, it is observed that the use of prophylactic agents has a different effect on the molecular composition of the dental biofilm in patients with different cariogenic conditions. Figure 3 and Figure 4 and Table 2 show that the use of prophylactic agents (toothpaste and tablets) in patients in the healthy (control) group led to a significant increase in the phosphate/protein/lipid ratio in biofilm samples relative to the value for stage I of the experiment (without prophylactics). For the carious group in the R1 study, the ratio was virtually unchanged with the use of toothpaste, and it decreased with application of a mineral complex containing calcium glycerophosphate.

Use of prophylaxis had no effect on the phosphate/mineral ratio for patients in the healthy group; however, the R2 ratio increased for participants with surface caries compared to stage I (without prophylaxis).

For the healthy group, a significant increase in the R3 ratio relative to stage I (without prophylactics) was observed for patients using toothpaste and tablets containing a mineral complex with calcium glycerophosphate; using tablets resulted in an almost 10-fold increase in the ratio. For patients in the carious group, the R3 ratio also increased with prophylactics, although not as much as for the healthy (control) group. Relative to the initial stage of the experiment, a greater increase in R3 was recorded with toothpaste than with tablets containing a mineral complex with calcium glycerophosphate.

Regarding the effect of prophylaxis type (exo/endo) for each participant group (healthy/carious), statistically significant differences at the *p* < 0.05 level were observed in the following cases, as shown in Table 3. For the healthy group, the differences were significant for the phosphate/protein/lipid ratio R1 and the phospholipid/lipid ratio R3 for both paste and tablets. For the carious group, differences at the *p* < 0.05 level were observed for only the R3 ratio for toothpaste, and for all three ratios for tablets.

From the analysis of variance, statistically significant intergroup differences between the healthy and carious groups (Table 4) were observed for the phosphate/protein/lipid ratio R1 for both toothpaste and tablets. For the phosphate/mineral R2 and phospholipid/lipid R3 ratios, significant intergroup differences between the healthy and carious groups were only observed for tablets. No significant intergroup differences were observed for R2 and R3 for toothpaste.

To understand the changes in the R1–R3 ratios between the organic and mineral components, and the significance of these changes with regard to the prophylaxis stage and the cariogenic conditions of the participants, we consider our previous study [27,40] on the use of exogenous and endogenous prophylaxis methods to saturate the oral fluid with ions necessary for remineralisation.

Oral hygiene with a toothpaste containing calcium glycerophosphate has been shown to briefly saturate the oral fluid with phosphates. Use of tablets containing a mineral complex with calcium glycerophosphate results in a long-term presence of mineral groups and complexes in the oral fluid. Thus, it is clear that an increase in the R1 phosphate/protein/lipid ratio in biofilm samples of the control (healthy) group with prophylactic agents is related to both the saturation of the biofilm with mineral complexes and the microbiota condition. A higher concentration of phosphate in the biofilm indicates a higher potential for preventing acid attacks [50].

The phosphate/mineral R2 ratio does not change in patients in the healthy group depending on the stage of prophylaxis, indicating a normal balance between different phosphate derivatives entering the biofilm. The phosphate/mineral R2 ratio increases significantly in the carious group when prophylactic agents are used. In our opinion, this may be related to a decrease in the proportion of carbs present in the mouth of those with advanced dental caries during the prophylaxis stage. In the IR-spectrum, the oscillations of carbs–hydrates overlap with oscillations of the mineral component, which is reflected in the reduced integral intensity in the 1110–960 cm^−1^ region. The increase in R2 ratio at stage III (administration of tablets containing mineral complex with glycerophosphate) indicates the predominance of glycerophosphates in the mineral component, a violation of the balance of phosphorus derivatives adsorbed by the biofilm. The pattern of changes in the phospholipid/lipid R3 ratio depending on prophylaxis stage and cariogenic conditions is similar to that observed for the R1 ratio.

Changes in R1–R3 values and the presence of statistically significant intra- and intergroup differences in R1–R3 indicate that the mechanisms of ion adsorption, compounds and complexes arriving from oral fluid into the dental biofilm during exo/endogenous prophylaxis, differ in normal patients and caries development. In our opinion, the conformational environment and charge interaction in the microbiota and the electrostatic state of the biofilm protein network play important roles.

It is known that the Amide I band is very sensitive to the secondary structure of the protein; FTIR spectroscopy is frequently used to study protein conformation and aggregation processes in vitro [45,51]. Based on the observed shifts in the frequency of the secondary structure component of the Amide I band [40,41,45,52], the effect of different factors on protein conformation processes can be established. Analysing the IR spectroscopy data (Figure 4), the position and shape (half-width) of the high-frequency component of the Amide I band in the region from 1700 to 1600 cm^−1^ depend on the prophylaxis type and also on the cariogenic conditions of the patient. Normally (for healthy patients), the use of toothpaste and tablets leads to a shift in the Amide I band to the low-frequency part of the spectrum in comparison with stage I (without prophylaxis), and to a reduction in the half-width. The position shift and decrease in half-width of the line are significantly greater with tablets than with toothpaste, probably due to the residence time of the prophylactic components in the oral fluid and their interaction with the biofilm.

For the carious group in stage I of the experiment (without prophylaxis), the position of the high-frequency component of the Amide I band is already shifted in the infrared spectrum relative to that observed for the healthy group. With toothpaste, there is a shift in position and a decrease in the half-width of the Amide I band, which is similar to that observed for the healthy group (normal). However, with tablets containing a mineral complex with calcium glycerophosphate, a shift in the Amide I band and a decrease in the half-width of the line are not observed.

The spectroscopic data we obtained testify to the different conformational environments and secondary structure of dental biofilm proteins in patients with different cariogenic conditions. The observed shift in the maximum high-frequency component of the Amide I band and the decrease in the half-width are related to the redistribution of the intensity of the components of the protein secondary structure [41,45,53], namely, α-helix (1648–1641 cm^−1^) and α-helix (approximately 1660 cm^−1^). This is a consequence of the difference in the microbiota in normal cases and carious pathology [2], and the exposure of the biofilm to prophylactic agents [50].

In view of the multistage process of ion exchange, active organic–mineral agents included in prophylactics must be retained in the oral cavity for a significant period of time [26] to be adsorbed by the biofilm. The concentration of these components in the biofilm must exceed their concentration in the hydrate layer of enamel to ensure an effective process of diffusion to the enamel surface [9,17]. However, concentrations of calcium and phosphate in biofilm, saliva, or artificial calcium-containing products that are too high negate the effect of preventive measures [27]. High levels of phosphate and calcium lead to rapid deposition of calcium phosphate in mineral phases on the enamel surface, which prevents normal remineralisation [54]. A detectable increase in the concentration of phosphate ions in the biofilm, especially when using tablets containing a mineral complex with glycerophosphate, can cause deposition and formation of extraneous calcium phosphates on the enamel surface, as indicated by an increase in the phosphate–mineral ratio R2 in patients in the carious group. This can cause limited permeability through the biofilm, plaque build-up, increased enamel solubility, and subsequent development of caries.

From the results, existing oral preventive measures may be insufficient, and often harmful, without considering personalised cariogenic data, probably mainly because the microorganisms involved aggregate into complex biofilm communities with different functioning in normality and pathology compared with the functioning of planktonic cells [1,2,9,10]. Thus, biofilm control is fundamental to oral health and the prevention of caries, gingivitis, and periodontitis. Understanding changes in the molecular and phase composition of dental hard tissues, oral fluid, and dental biofilm with different oral homeostasis conditions will allow successful transition to a personalised approach in dentistry and high-tech healthcare, and effective caries prevention.

## 5. Limitations

This study has certain limitations connected with the number and age of participants of the study, and the spatial resolution of the diagnostics methods (FTIR spectroscopy) applied for the analysis of the features related to the molecular structure of dental biofilms.

## 6. Conclusions

Based on data obtained by synchrotron FTIR, calculation of organic and mineral component ratios, and statistical analysis of the data, we were able to assess changes occurring in the molecular composition of the dental biofilm in individuals with different cariogenic conditions.

Changes in the phosphate/protein/lipid, phosphate/mineral, and phospholipid/lipid ratios as well as the presence of statistically significant intra- and inter-group differences in these ratios indicate that the mechanisms of ion adsorption, compounds and complexes arriving from oral fluid into dental biofilm during exo/endogenous prophylaxis, differ for people with various cariogenic situation in oral cavity (norm/carious).

## 7. Recommendation of an Expert

Prophylaxis and prevention of dental caries is one of the main scientific directions in preventive dentistry. Receptivity to caries is known to be associated with the structure and properties of the dental tissue, the structure of dentitions and jaws, composition of the oral fluid, the level of performed personalised prophylaxis and the condition of the whole organism. The latest investigations show that the factors resulting in the emergence and development of the dental caries as well as the conditions favouring formation of caries resistance have a different nature and they require long-term and accurate examination.

However, a preconceived paradigm implies that reducing caries prevalence in the population can be attained due to the personalised diagnostics as well the effective prevention of the disease. It means that remineralisation therapy is required—compulsory saturation of the dental tissue with mineral compounds, realised with the use the agents that are present in the oral fluid when performing exo- and endogenous preventive measures.

The conditions providing these physicochemical depend on the composition and properties of the oral fluid interacting with the surface layer of enamel through the complex multi-layered structure formed on enamel, i.e., biofilm. Although dental plaques cannot be completely removed, pathogenic properties of biofilm can be reduced in a dependence on cariogenic state of a patient by diminishing the biological load and keeping f the corresponding bio-balance, using exo- and endogenous prevention techniques.

Activation of the studies in this area and a deeper comprehension of the dental bio-film role in a dependence on homeostasis conditions within the oral cavity will have a considerable impact on the clinical practice in future. It enables effective caries prevention and makes the transfer to a personalised approach in dentistry, as well as in high-tech public healthcare.

## Figures and Tables

**Table 1:** Active vibrations in infrared absorption spectra of dental biofilm samples.

Molecular Group Assignment	Vibration Modes	Wave Numbers, cm^−1^	References
Proteins (α-Amylase; Albumin; Cystatins; Mucins; Proline-rich proteins; sIgA) -HormonesBREAK (Cortisol; Testosterone)	O-H str of hydroxyl groups	3600–3100	[28,29,42]
functional groups dominated by fatty acid chains, lipids (phospholipids)	C–H str (asym) of –CH_3_, C–H str (asym) of > CH_2_,	2963–2855	[28,29,42,43,48]
Lipid ester carbonyl and DNA strands	>C=O stretching, C=O stretching groups and DNA characteristic of base-paired	1740–1710	[28,29,43,48,49]
Protein, Peptide	Amide I of α-helical structures, Amide I C=O stretching and Amide II N–H bending	1675–1615	[28,29,41,42,43,45]
Proteins, tryptophan, Peptide	Amide II, νN–H, νC=N stretching, νC=C	1575–1520	[28,29,42,43]
Amino acid side chains, lipids and proteins	Asymmetric CH_2_ bending Methyl bending of	1469–1455	[28,29,41,42,43,44,45]
Fibrinogen/methyl bending of amino acid side chains, lipids and proteins	Symmetric CH_3_ bending, Stretching of COO	1412–1396	[28,29,41,42,43,44,45]
Proteins	Amide III coupled N–H/C–H deformations, τ (N–H), ν (C–N), τ (C=O), ν (C–C), ν (CH_3_)	1350–1200	[28,29,42,43]
Phosphodiester groups in DNA, Proteins (amide III, mainly α–helix conformation)	P=O of PO_2_^−^ stretching, Amide III Asymmetric C–N stretching	1250–1240	[28,29,42,43,44]
Ester; Membrane lipids (phospholipids); Carbohydrates	P=O of PO_2_, CO-O-C antisymmetric stretching C–O, C–C stretching and C–O–H, C–O–C deformation	1171–1160	[28,29,42,43,44]
Polysaccharides, Carbohydrates, Phosphates, glycerophosphate and phosphatase; Phospholipids Phosphodiester groups in DNA	PO_2_^−^ stretching, CH_2_ OH groups, C–O stretching and COH groups bending, C–C, C–O–P–O–C	1085–1050	[28,29,42,43,44]
Phosphodiester residue (DNA)	C–O–P	975–960	[29,42,43,44]
Carbohydrates and (CH3)3 symmetric stretching Membrane lipids (phospholipids)	C–O, C–C stretching, C–O–H, C–O–C deformation C–H stretch, 3rd overtones	929–924	[29,42]
Fingerprint region	Anomeric ring vibrations for tryptophan, tyrosine, and phenyloalanine	900–800	[29,42]

**Table 2:** Calculated R1–R3 ratios (mean ± standard deviation).

Participants in Experiment	Ratio	Stage of Experiment		
		I Without Prophylaxis	II Toothpaste	III Tablet
Healthy group	R1 phosphate/protein/lipid	1.21 ± 0.20	2.23 ± 0.368	4.88 ± 0.68
	R2 phosphate/mineral	0.06 ± 0.008	0.05 ± 0.008	0.06 ± 0.01
	R3 phospholipid/lipid	0.09 ± 0.016	0.35 ± 0.048	1.31 ± 0.22
Caries group	R1 phosphate/protein/lipid	1.05 ± 0.14	1.20 ± 0.21	0.54 ± 0.086
	R2 phosphate/mineral	0.05 ± 0.008	0.08 ± 0.011	0.13 ± 0.018
	R3 phospholipid/lipid	0.06 ± 0.011	0.59 ± 0.105	0.27 ± 0.038

**Table 3:** Intragroup differences for R1–R3 ratios according to prophylaxis type in relation to initial stage (stage I). “+”, statistically significant differences at the *p* < 0.05 significance level; “−”, no statistically significant differences. Significance level according to the test is shown in brackets.

Participant Group	Stages of Experiment (Stages of Prophylaxis)	Ratios		
		R1	R2	R3
Healthy	I (without prophylaxis)—II (toothpaste)	+ [0.035]	−	+ [0.005]
	I (without prophylaxis)—III (tablet)	+ [0.01]	−	+ [0.007]
Carious	I (without prophylaxis)—II (toothpaste)	−	−	+ [0.003]
	I (without prophylaxis)—III (tablet)	+ [0.01]	+ [0.005]	+ [0.001]

**Table 4:** Intergroup differences in R1–R3 ratios (between healthy and carious participant groups) depending on type of prophylaxis. “+”, statistically significant differences at the *p* < 0.05 significance level; “−”, no statistically significant differences. Significance level according to the test is shown in brackets.

Stages of Experiment (Stages of Prophylaxis)	Ratios		
	R1	R2	R3
II (toothpaste)	+ [0.01]	−	−
III (tablet)	+ [0.003]	+ [0.01]	+ [0.001]

## Data Availability

The data that support the findings of this study are available from the corresponding author upon reasonable request.
